# Prevalence, antimicrobial resistance, and virulence factors of *Enterococcus* spp. infecting broiler chickens in Egypt

**DOI:** 10.1038/s41598-026-62799-3

**Published:** 2026-07-23

**Authors:** Haitham Helmy Sayed, Reem M. Alsaadawy, Mahmoud Sabra, Shimaa El-Nagar, Waleed Younis

**Affiliations:** 1 Department of Microbiology and Immunology, Faculty of Veterinary Medicine, Sohag University, Sohag, 82524 Egypt; 2 Department of Zoonoses, Faculty of Veterinary Medicine, Assiut University, Assiut, 71526 Egypt; 3 Department of Poultry Diseases, Faculty of Veterinary Medicine, Qena University, Qena, 83523 Egypt; 4 Reference Laboratory for Veterinary Quality Control on Poultry Production (RLQP), Animal Health Research Institute, Luxor, Egypt; 5 Department of Microbiology, Faculty of Veterinary Medicine, Qena University, Qena, 83523 Egypt

**Keywords:** Antimicrobial resistance, Broiler chickens, Egypt, *Enterococcus* spp., Prevalence, Virulence factors, Diseases, Microbiology

## Abstract

*Enterococcus* spp. are potential pathogens in broiler chickens, causing economic losses in addition to their extensive capacity to acquire and disseminate antimicrobial resistance genes among various bacterial species. This study was conducted to investigate the occurrence of enterococcal infections among broiler chickens in Qena and Sohag Governorates, Egypt, to determine antimicrobial resistance and virulence factors of enterococci isolates, and to screen them for the presence of class 1 and 2 integrons by PCR. Therefore, 120 samples were collected from the diseased broiler chickens aged 1 to 35 days from 30 different commercial farms. The birds were clinically and bacteriologically examined for enterococci, and then antimicrobial susceptibility and virulence factors of enterococci isolates were determined, in addition to their investigation for the presence of some genes by PCR. Enterococci were isolated from 22.5% of the examined birds and were identified as *E. faecalis*, *E. faecium*, and *E. gallinarum* with prevalence of 11.7%, 7.5%, and 3.3%, respectively. The infected birds showed lameness, body weight loss, arthritis, omphalitis, and yolk sac infection in chicks, as well as septicemia and endocarditis in some birds. All enterococci isolates were gelatinase producers, while 18.5% and 81.5% of them were β-haemolytic and biofilm formers with varying grades, respectively. Respective to isolatesʼ antimicrobial susceptibility, 63% of them were multidrug-resistant enterococci with higher occurrence (70.6%) among the class 1 integrons-positive isolates. PCR revealed that 100%, 81.5%, 74.1%, 74.1%, 48.1%, 37.0%, 7.4%, and 3.7% of the isolates carried *gelE*, *aac(6’)/aph(2’’)*, *ermB*, *tetM*, *int1*, *cylA*, *vanA*, and *int2* genes, respectively. This study demonstrated a high prevalence of pathogenic MDR-enterococci among broiler chickens, posing a hazard for poultry health and a potential public health concern. Furthermore, it reported for the first time, to the best of our knowledge, the presence of a high prevalence of class 1 integrons among enterococci in Egypt. Therefore, strict biosecurity protocols and prudent antimicrobial stewardship strategies should be implemented urgently.

**Supplementary Information:**

The online version contains supplementary material available at 10.1038/s41598-026-62799-3.

## Introduction

The poultry industry is considered one of the main sources of the national income in Egypt, and it contributes to achieving food security^1^. Broiler production represents the largest element of the Egyptian poultry industry, where it covers about 95% of the country’s total poultry consumption^2^. The immune system and intensive production of broilers are challenged by many disorders, infections, heat stress, and stress caused by management that can result in economic losses^3,4^.

*Enterococcus* spp. are gram-positive cocci, catalase-negative, and facultative anaerobes^5^. They are ubiquitous and constitute a part of the gastrointestinal flora of mammals, birds, insects, and reptiles^6^. Enterococci are particularly challenging to eliminate due to their ability to adapt to environmental stressors^7^. Although enterococci are often viewed as commensal organisms, they are opportunistic pathogens that can cause severe infections and possess a strong capacity to acquire, express, and transfer antimicrobial resistance genes (AMRGs)^8,9^.

*Enterococcus* spp. are considered one of the common pathogens causing economic losses in poultry production, where they cause growth retardation, relatively high mortality rates, increased condemnations in the slaughterhouse, in addition to the cost of prevention and control of the infections^10^. They have been implicated in a variety of pathologies, including arthritis, osteomyelitis, lameness, and endocarditis^11^, mortality in the first week, pulmonary hypertension, encephalomalacia, and neurologic disorders^12^. Enterococcal outbreaks are considered opportunistic, while predisposing factors are deemed crucial to the outcome of the pathology induced^13^.

Poultry and its products are the most consumed worldwide, and they can be contaminated with enterococci, posing a risk to human health if consumed^14^. Due to the occurrence, prevalence, and persistence of enterococci throughout the one health continuum, they are a major candidate for a one health approach-based investigation of zoonotic infections, including antimicrobial resistance (AMR)^6,15^. Enterococci are becoming significant pathogens worldwide and are one of the most common causes of nosocomial infections, and are likely to continue acquiring antibiotic resistance^16^.

Enterococci can be involved in urinary tract infections, bacteriemia, peritonitis, endocarditis, wound infections, intra-abdominal and pelvic infections, catheter infections, surgical infections, and central nervous system infections^17^. According to Abu-Gharbia et al.^18^, enterococci are important uropathogens as they are one of the most common causes of urinary tract infections. *E. faecalis* and *E. faecium* are the most important enterococci, causing about 90% of the clinical infections in humans; however, other *Enterococcus* spp. have been isolated from patients with a variety of infections^17,19,20^.

For bacteria to be pathogenic, they must possess virulence factors (VFs) in addition to AMR. Many VFs are involved in the pathological process of *Enterococcus* spp.^21^. Cytolysin, gelatinase, and hyaluronidase encoded by *cylA*, *gelE*, and *hyl* genes, respectively, are the most frequent VFs of *Enterococcus* spp. affecting host cells with their toxic or destructive effects^22^. Likewise, biofilm formation (BF) is considered a fundamental factor in the pathogenesis of several opportunistic bacteria, as in enterococcal infections^23^.

AMR is considered a one-health problem affecting humans, animals, and the environment worldwide^6^. Antimicrobial usage is considered a major factor in the development, selection, and spreading of antibiotic-resistant bacteria^24^. Human practices, such as the excessive use of antimicrobials in poultry farms for prophylaxis, treatment, and as growth promoters, generate chains of AMR^15,25^ and raise the risk of developing hotspots for zoonotic disease resurgence^15^.

Recently, antimicrobial-resistant enterococci isolated from poultry farms have become a problem in many countries^26^, and multidrug-resistant (MDR) enterococci have become common^10^. Emergence of drug-resistant and MDR-enterococci in poultry is of major concern, as they limit the therapeutic options for the infections, in addition to the possibility of the dispersion between poultry and humans and transfer of AMRGs to other species of bacteria^14^.

*Enterococcus* spp. are characterized by relatively rapid acquisition and spread of AMRGs through Mobile genetic elements (MGEs)^12^. Class 1 and 2 integrons play an important role in the dissemination of AMRGs among the different species of bacteria and may contribute to the development of MDR bacteria^27^. Most of the studies about integrons were performed in gram-negative bacteria, with only a few exceptions, although in recent years, integrons have been identified as a novel and unexpected antibiotic determinant in gram-positive bacteria^28^; therefore, it is of great interest to search for the role of class 1 and 2 integrons in AMR among enterococci.

A variety of *Enterococcus* spp., rising in prevalence, AMR, VFs, and associated diseases that impact poultry production, have been described worldwide^29^. However, to the best of our knowledge, limited studies^10,30–34^ have focused on Enterococcal infections in broiler chickens in Egypt and about AMR as well as VFs of enterococci isolated from them, and none of these studies was performed in Qena and Sohag Governorates, Egypt. Furthermore, this is the first study about the prevalence of class 1 and 2 integrons among enterococci isolated from broiler chickens in Egypt.

Results of such studies are essential to determine the importance of Enterococcal infections in broiler chickens and to take suitable measures for their prevention and control, in addition to their public health significance. Considering the above facts, this study aimed to investigate the occurrence of Enterococcal infections among broiler chickens at Qena and Sohag Governorates, Egypt, through clinical and bacteriological examinations, determine AMR and VFs of the isolates, and to screen them for the presence of class 1 and 2 integrons by PCR.

## Materials and methods

### Sampling and clinical examination

This study was performed on 120 samples from the diseased broiler chickens aged 1 to 35 days. These samples were randomly collected from 30 different farms (4 samples per farm) and were randomly selected during the routine clinical and post-mortem examinations of the diseased broiler chickens obtained from the various commercial farms in Qena and Sohag Governorates, Egypt. For the clinical examination of the diseased broilers, the birds were anaesthetized using a ketamine intramuscular injection (5–10 mg/kg), prior to being sacrificed by cervical dislocation. All animal operations complied with the approved animal ethics protocol. Samples were collected from the yolk sac, heart, liver, lung, spleen, and joints under aseptic conditions for bacteriological examination for enterococci.

### Isolation and identification of enterococci

Samples were inoculated into Brain Heart Infusion broth (BHI broth) (Oxoid, UK) and incubated aerobically at 37 °C for 24 h. Then, a loopful of the inoculated broth was subcultured on bile esculin azide agar (Oxoid, UK) and incubated aerobically at 37 °C for 24–48 h^10^. The suspected isolates were initially identified through morphological characteristics, Gram-staining, catalase, and oxidase tests according to Quinn et al.^35^, then they were identified by the VITEK 2 compact system using Gram-positive identification cards (bioMérieux, France) according to the manufacturer’s instructions.

### Determination of some virulence factors of enterococci isolates

#### Haemolytic activity

The tested enterococci isolates were plated on Blood agar base (Oxoid, UK) supplemented with 5% defibrinated sheep blood, then incubated aerobically at 37 °C for 24 h. A positive result was indicated by the appearance of clear zones around the colonies^36^.

#### Gelatinase activity

The tested enterococci isolates were plated on Trypticase soya agar (Oxoid, UK) containing 3% gelatine (Oxoid, UK). The inoculated plates were incubated aerobically at 37 °C for 24 h, then preserved in a refrigerator at 4 °C for 30 min. A positive result was indicated by the appearance of clear zones around the colonies^37^.

#### Biofilm formation

The ability of enterococci isolates for BF was investigated by Microtiter plate assay as described by Stępień-Pyśniak et al.^21^, with slight modification. The tested isolates were plated on Columbia agar base (Oxoid, UK) supplemented with 5% defibrinated sheep blood and incubated aerobically at 37 °C for 24 h. After that, a few colonies of identical morphology from each isolate were suspended in physiological saline, and the bacterial suspension was adjusted to a 0.5 McFarland standard (10^8^ CFU/ml).

Each isolate was tested in quadruplicate by transferring 20 µl of the bacterial suspension to 2 wells of a polystyrene microtitre plate (Costar, USA) containing 180 µl of BHI broth supplemented with 2% glucose (Oxford, India) and 2 wells of another microtitre plate containing 180 µl of Tryptic Soy Broth (Oxoid, UK) supplemented with 1% glucose. Eight wells were used as a negative control for each plate; they contained 200 µl of each broth supplemented with glucose only. After 24 h of incubation aerobically at 37◦C, the wells’ contents were carefully removed and gently washed with phosphate-buffered saline three times, then the plates were left in an inverted position at room temperature overnight. Later, 200 µl of 0.1% crystal violet (Fluka, Germany) was added to each well for 15 min, then it was removed, and the excess stain was rinsed off with running tap water until the washing was free from the stain. After drying the plates at room temperature, 200 µl of 96% ethanol (Piochem, Egypt) was added to each well for 30 min without shaking.

Optical densities of the stained adherent biofilms (OD) were measured with a microplate reader (BioTek ELX800, USA) at 620 nm three times; the mean OD for each tested isolate was calculated, and the cut-off OD (ODc) was considered as three standard deviations above the mean OD of the negative control. Based on OD, enterococci isolates were classified as non-BFs when OD < ODc, weak BFs when ODc < OD < 2ODc, moderate BFs when 2ODc < OD < 4ODc, and as strong BFs when OD > 4ODc.

### Antimicrobial susceptibility testing for enterococci isolates

Using the Kirby-Bauer disk diffusion method, antimicrobial susceptibility of enterococci isolates was tested against 15 different antimicrobial agents (Bioanalyse, Turkey) (Table 1). These antimicrobials were selected based on CLSI guidelines for *Enterococcus* spp. and on their significance for clinical use. Each tested isolate was streaked on two plates of Mueller-Hinton agar (Oxoid, UK) supplemented with 5% sheep RBCs. Then, the used antimicrobials were dispensed on the inoculated plates and incubated at 37 °C for 24 h. Later, inhibition zones diameters were measured, and the results were interpreted according to CLSI^38^ when the breakpoints of antimicrobials are available, otherwise, based on breakpoints for the other antimicrobials from previous literature, as illustrated in Table 1. *S. aureus* ATCC 25,923 and E. *faecalis* ATCC 29,212 were used as quality controls. Resistance of the isolate for three Antimicrobials of different classes or more was interpreted as MDR^39^.

**Table 1:** Antimicrobials and zone diameter breakpoints used in this study to determine antimicrobial susceptibility of enterococci isolates.

Antimicrobial class	Antimicrobial agent	Disc concentration (µg)	Zone diameter breakpoints (mm)			References
			Resistant	Intermediate	Sensitive	
Β-lactams	AM	10	≤ 16	-	≥ 17	^38^
	AX	30	≤ 16	-	≥ 17	^78^
	AMC	30	≤ 13	14–17	≥ 18	^79^
	CXM	30	≤ 10	11–19	≥ 20	^80^
Glycopeptides	VA	30	≤ 14	15–16	≥ 17	^38^
Macrolides	E	15	≤ 13	14–22	≥ 23	^38^
Aminoglycosides	CN	10	≤ 12	13–14	≥ 15	^79^
	S	10	≤ 11	12–14	≥ 15	^79^
Quinolones	CIP	5	≤ 15	16–20	≥ 21	^38^
	ENR	5	≤ 15	16–20	≥ 21	^81^
Phenicols	C	30	≤ 12	13–17	≥ 18	^38^
Tetracyclines	T	30	≤ 14	15–18	≥ 19	^38^
	DO	30	≤ 12	13–15	≥ 16	^38^
Lincosamides	L	2	≤ 8	9–11	≥ 12	^82^
Phosphonic acid derivatives	FF	200	≤ 12	13–15	≥ 16	^38^
Sulfonamides	SXT	25	≤ 10	11–15	≥ 16	^79^

### Detection of some genes in enterococci isolates by PCR

#### DNA extraction

After growth of enterococci isolates on BHI broth overnight at 37 °C, bacterial DNA extraction was performed using QIAamp DNA Mini Kit (Qiagen GmbH, Germany) according to the manufacturer’s instructions.

#### DNA amplification

Amplification of DNA was performed in a T3 thermocycler (Biometra, Germany) for the detection of *aac*(6’)/*aph*(2’’), *ermB*, *tetM*, *vanA*, *int1*, *int2*, *cylA*, and *gelE* genes in enterococci isolates using the oligonucleotide primers (Metabion, Germany) illustrated in Table 2 and Emerald Amp Max PCR Master Mix (Takara, Japan) under PCR conditions illustrated in Table 2 for each target gene. The reaction mixture was prepared in a 25 µl mixture according to the manufacturer’s instructions, containing 12.5 µl from Master Mix, 5 µl from extracted DNA, 1 µl from each of forward and reverse primers (20 pmol), and 5.5 µl from nuclease-free water.

**Table 2:** The target genes in the study, oligonucleotide primers, and PCR conditions used.

Target gene	Primers sequences (5`- 3`)	Product size (bp)	Primary denaturation	Number of cycles (No.) and PCR conditions				Final extension	References
				No.	Denaturation	Annealing	Extension		
*aac(6’)/* *aph(2’’)*	F-GAAGTACGCAGAAGAGA	491	94 ˚C 5 min.	35	94 ˚C 30 s.	54 ˚C 40 s.	72 ˚C 40 s.	72 ˚C 10 min.	^33^
	R-ACATGGCAAGCTCTAGGA								
*ermB*	F-CATTTAACGACGAAACTGGC	405	94 ˚C 3 min.	35	94 ˚C 30 s.	55 ˚C 30 s.	72 ˚C 1 min.	72 ˚C 5 min.	^83^
	R-GGAACATCTGTGGTATGGCG								
*tetM*	F-GTGGACAAAGGTACAACGAG	406	95 ˚C 5 min.	30	95 ˚C 1 min.	62 ˚C 1 min.	72 ˚C 1 min.	72 ˚C 7 min.	^84^
	R-CGGTAAAGTTCGTCACACAC								
*vanA*	F-CATGACGTATCGGTAAAATC	885	95 ˚C 10 min.	36	94 ˚C 1 min.	56 ˚C 1 min.	74 ˚C 1 min.	74 ˚C 10 min.	^85^
	R-ACCGGGCAGRGTATTGAC								
*int1*	F-CCTCCCGCACGATGATC	280	96 ˚C 5 min.	30	96 ˚C 30 s.	55 ˚C 30 s.	72 ˚C 30 s.	72 ˚C 7 min.	^75^
	R-TCCACGCATCGTCAGGC								
*int2*	F-TTGCGAGTATCCATAACCTG	288	94 ˚C 5 min.	35	94 ˚C 30 s.	55 ˚C 30 s.	72 ˚C 1 min.	72 ˚C 7 min.	^62^
	R-TTACCTGCACTGGATTAAGC								
*cylA*	F-ACTCGGGGATTGATAGGC	688	95 ˚C 15 min.	30	94 ˚C 1 min.	56 ˚C 1 min.	72 ˚C 1 min.	72 ˚C 10 min.	^86^
	R-GCTGCTAAAGCTGCGCTT								
*gelE*	F-TATGACAATGCTTTTTGGGAT	213	95 ˚C 15 min.	30	94 ˚C 1 min.	56 ˚C 1 min.	72 ˚C 1 min.	72 ˚C 10 min.	
	R-AGATGCACCCGAAATAATATA								

### Analysis of PCR products

Products of PCR were electrophoresed on a 1.5% agarose gel (Biometra, Germany) using gradients of 5 V/cm at room temperature and a general 100 bp DNA ladder (Thermo Fisher Scientific, Lithuania) to determine the fragment sizes. Gel was photographed using a gel documentation system (Alpha Innotech, USA).

### Statistical analysis

In this study, prevalence, VFs, and AMR of *Enterococcus* spp, as well as the distribution of the investigated genes among them, were all represented as percentages. Phenotypic-genotypic relationship of AMR and virulence for the isolates, the association between AMR and BF, as well as the association between AMR and the presence of integrons, were all assessed by Fisher’s exact test using SPSS version 22, and a P value ˂ 0.05 was statistically significant.

## Results

### Clinical and post-mortem findings

The infected broiler chickens showed depression, lethargy, ruffled feathers, lameness, and body weight loss. Post-mortem examination revealed arthritis, septicemia, and endocarditis in some birds as well as omphalitis and yolk sac infection in the infected chicks.

### Prevalence of enterococcus spp. among the examined broiler chickens

Out of the examined samples, enterococci were isolated and phenotypically identified from 27 samples with a total prevalence of 22.5%. On bile esculin azide agar, enterococci produced small transparent colonies surrounded by a brown-black zone. They were catalase-negative, oxidase-negative, and appeared as gram-positive cocci arranged in pairs or short chains on microscopical examination. By the VITEK 2 system, the isolates were identified as *E. faecalis* (*n* = 14: F1-F14), followed by *E. faecium* (*n* = 9: C1-C9) and *E. gallinarum* (*n* = 4: G1-G4), with prevalence of 11.7%, 7.5%, and 3.3% among the examined broiler chickens, respectively.

### Virulence factors and genes of enterococci isolates

As illustrated in Tables 1 and 3 8.5% of enterococci isolates produced β-haemolysis on blood agar, while 37% of them harbored the *cylA* gene (Supplementary Fig. 1), and statistical analysis revealed that there was a significant difference in β-haemolytic activity-*cylA* gene association (*P* = 0.001). On the other hand, all enterococci isolates exhibited gelatinase activity and harbored *gelE* gene (Supplementary Fig. 2), while only 81.5% of them were biofilm formers (BFs) with varying grades as illustrated in Table 4.

**Table 3:** Virulence factors and genes of isolated *Enterococcus* spp.

Enterococcus spp.	Haemolytic activity		cylA gene		Gelatinase activity		gelE gene		Biofilm formation	
	No.	%	No.	%	No.	%	No.	%	No.	%
*E. faecalis* (n = 14)	4	28.6	6	42.9	14	100	14	100	12	85.7
*E. faecium* (n = 9)	1	11.1	3	33.3	9	100	9	100	8	88.9
*E. gallinarum* (n = 4)	0	0	1	25	4	100	4	100	2	05
Total (n = 27)	5	18.5	10	37.0	27	100	27	100	22	0.581
P value	< 0.001^*^				–					
					–					

**Table 4:** Biofilm formation grades by isolated *Enterococcus* spp.

Enterococcus spp.	Non-biofilm formers		Weak biofilm formers		Moderate biofilm formers		Strong biofilm formers		Total of biofilm formers	
	No.	%	No.	%	No.	%	No.	%	No.	%
*E. faecalis* (n = 14)	2	14.3	2	14.3	6	42.8	4	28.6	12	85.7
*E. faecium* (n = 9)	1	11.1	0	0	5	55.6	3	33.3	8	88.9
*E. gallinarum* (n = 4)	2	05	0	0	2	05	0	0	2	05
Total (n = 27)	5	18.5	2	7.4	13	48.2	7	25.9	22	0.581

### Antimicrobial resistance of enterococci isolates

Table 5 illustrates the antimicrobial susceptibility of enterococci isolates. They were sensitive to vancomycin (VA) (100%), followed by amoxicillin/clavulanic acid (AMC) (92.6%), enrofloxacin (ENR) (92.6%), amoxicillin (AX) (88.9%), ciprofloxacin (CIP) (88.9%), ampicillin (AM) (85.2%), fosfomycin (FF) (81.5%), cefuroxime (CXM) (77.8%), trimethoprim/sulphamethoxazole (SXT) (70.4%), and chloramphenicol (C) (66.7%); while were resistant to lincomycin (L) (66.7%) and tetracycline (T) (66.7%), followed by erythromycin (E) (63%), doxycycline (DO) (59.3%), streptomycin (S) (55.6%), and gentamicin (CN) (51.9%). Furthermore, 63% of the isolates were characterized as MDR-enterococci with many different antimicrobial resistance profiles (AMRPs) as illustrated in Table 6. Statistical analysis revealed that there were no significant differences in the phenotypic AMR or MDR-BF association except in AMR of T and SXT-BF association, as illustrated in Supplementary Tables 1 and 2.

**Table 5:** Results of antimicrobial susceptibility of enterococci isolates.

Antimicrobials	*E. faecalis* (*n* = 14)						*E. faecium* (*n* = 9)						*E. gallinarum* (*n* = 4)						Total (*n* = 27)					
	S.		I.		*R*.		S.		I.		*R*.		S.		I.		*R*.		S.		I.		*R*.	
	*N*.	%	*N*.	%	*N*.	%	*N*.	%	*N*.	%	*N*.	%	*N*.	%	*N*.	%	*N*.	%	*N*.	%	*N*.	%	*N*.	%
AM	10	71.4	2	14.3	2	14.3	6	66.7	1	11.1	2	22.2	4	100	0	0	0	0	20	74.1	3	11.1	4	14.8
AX	12	85.8	1	7.1	1	7.1	7	77.8	0	0	2	22.2	4	100	0	0	0	0	23	85.2	1	3.7	3	11.1
AMC	13	92.9	0	0	1	7.1	7	77.8	1	11.1	1	11.1	4	100	0	0	0	0	24	88.9	1	3.7	2	7.4
CXM	8	57.1	4	28.6	2	14.3	6	66.7	0	0	3	33.3	2	50	1	25	1	25	16	59.3	5	18.5	6	22.2
VA	14	100	0	0	0	0	9	100	0	0	0	0	4	100	0	0	0	0	27	100	0	0	0	0
E	3	21.4	2	14.3	9	64.3	1	11.1	2	22.2	6	66.7	0	0	2	50	2	50	4	14.8	6	22.2	17	63
CN	5	35.7	2	14.3	7	50	2	22.2	2	22.2	5	55.6	1	25	1	25	2	50	8	29.6	5	18.5	14	51.9
S	3	21.4	3	21.4	8	57.1	2	22.2	2	22.2	5	55.6	1	25	1	25	2	50	6	22.2	6	22.2	15	55.6
CIP	10	71.4	2	14.3	2	14.3	6	66.7	2	22.2	1	11.1	3	75	1	25	0	0	19	70.4	5	18.5	3	11.1
ENR	10	71.4	2	14.3	2	14.3	6	66.7	3	33.3	0	0	3	75	1	25	0	0	19	70.4	6	22.2	2	7.4
C	5	35.7	4	28.6	5	35.7	5	55.6	2	22.2	2	22.2	1	25	1	25	2	50	11	40.8	7	25.9	9	33.3
T	2	14.3	3	21.4	9	64.3	1	11.1	1	11.1	7	77.8	1	25	1	25	2	50	4	14.8	5	18.5	18	66.7
DO	4	28.6	2	14.3	8	57.1	2	22.2	1	11.1	6	66.7	1	25	1	25	2	50	7	25.9	4	14.8	16	59.3
L	3	21.4	1	7.1	10	71.4	2	22.2	2	22.2	5	55.6	0	0	1	25	3	75	5	18.5	4	14.8	18	66.7
FF	8	57.1	4	28.6	2	14.3	4	44.5	3	33.3	2	22.2	2	50	1	25	1	25	14	51.9	8	29.6	5	18.5
SXT	7	50	3	21.4	4	28.6	5	55.6	1	11.1	3	33.3	3	75	0	0	1	25	15	55.6	4	14.8	8	29.6

**Table 6:** Antimicrobial resistance profiles of enterococci isolates and prevalence of class 1 and 2 integrons among them.

Isolate No.	Phenotypic antimicrobial resistance profiles	Genotypic antimicrobial resistance profiles				MDR	Class 1 integrons	Class 2 integrons
		aac(6’) /aph(2’’)	ermB	tetM	vanA			
F1	E, CN, S	+	+	***-***	***-***	Non-MDR	-	**-**
F2	E, C, T, DO, L, SXT	+	***+***	+	***-***	MDR	+	**-**
F3	AM, AX, AMC, CXM, E, CN, S, CIP, ENR, C, T, DO, L, FF, SXT	+	+	+	***-***	MDR	+	**+**
F4	E, CN, S, L	+	+	***-***	***-***	MDR	+	**-**
F5	E, T, DO	+	+	+	***-***	Non-MDR	+	**-**
F6	E, C, T, DO, L	+	+	+	***-***	MDR	+	**-**
F7	CN, S, T, DO, L	+	***-***	+	***-***	MDR	-	**-**
F8	E	+	+	+	***-***	Non-MDR	-	**-**
F9	AM, CXM, E, CIP, ENR, C, T, L, FF, SXT	+	+	+	***-***	MDR	+	**-**
F10	CN, S, T, DO, L	+	***-***	+	***-***	MDR	-	**-**
F11	T, DO, C, L, SXT	+	+	+	***-***	MDR	+	**-**
F12	CN, S	+	***-***	***-***	***-***	Non-MDR	-	**-**
F13	CN, S, L	+	***-***	***-***	***-***	Non-MDR	-	**-**
F14	E, S, T, DO, L	+	+	+	***-***	MDR	+	**-**
Total for *E. faecalis* isolates (n = 14)		100 (n = 14)	71.4 (n = 10)	71.4 (n = 10)	0 (n = 0)	64.3 (n = 9)	57.1 (n = 8)	7.1 (n = 1)
C1	AM, AX, AMC, CXM, E, CN, S, CIP, C, T, DO, L, FF, SXT	+	+	+	***-***	MDR	+	**-**
C2	E, CN, S, T, DO	+	+	+	***+***	MDR	+	**-**
C3	CXM, E, T, DO, L, SXT	+	+	+	***-***	MDR	+	**-**
C4	T	+	***-***	***-***	***-***	Non-MDR	-	**-**
C5	AM, AX, CXM, E, CN, S, C, T, DO, L, FF, SXT	+	+	+	***-***	MDR	+	**-**
C6	CN, S	+	***-***	***-***	***-***	Non-MDR	-	**-**
C7	E, T, DO, L	***-***	+	+	***-***	MDR	-	**-**
C8	E, CN, S, T, DO	+	+	+	***-***	MDR	-	**-**
C9	L	***–***	***–***	***–***	***-***	Non-MDR	-	**-**
Total for *E. faecium* isolates (n = 9)		77.8 (n = 7)	66.7 (n = 6)	66.7 (n = 6)	11.1 (n = 1)	66.7 (n = 6)	44.4 (n = 4)	0 (n = 0)
G1	CXM, E, C, T, DO, L, FF, SXT	***-***	+	+	+	MDR	+	**-**
G2	CN, S, T, DO, L	+	+	+	***-***	MDR	-	**-**
G3	C, L	***-***	+	+	***-***	Non-MDR	-	**-**
G4	E, CN, S	***-***	+	+	***-***	Non-MDR	-	**-**
Total for *E. gallinarum* isolates (n = 4)		25 (n = 1)	100 (n = 4)	100 (n = 4)	25 (n = 1)	50 (n = 2)	25 (n = 1)	0 (n = 0)
Total for enterococci isolates (n = 27)		81.5 (n = 22)	74.1 (n = 20)	74.1 (n = 20)	7.4 (n = 2)	63 (n = 17)	48.1 (n = 13)	3.7 (n = 1)

On the other hand, as illustrated in Table 6 and 81.5%, 74.1%, 74.1%, and 7.4% of enterococci isolates harbored *aac*(*6’*)*/aph*(2′′), *ermB*, *tetM*, and *vanA* genes, respectively (Supplementary Figs. 3–6), and various combinations of the investigated genes were found in most of the isolates. *ErmB* and *tetM* genes were reported together in 66.7% of the isolates, and statistical analysis revealed a significant difference in their association (*P* = 0.001), as illustrated in Supplementary Table 3.

On comparison of the phenotypic and genotypic AMRPs of enterococci isolates illustrated in Table 6, it was found that 100%, 40.9%, 36.4%, 20%, 15%, and 15% of *vanA*, *aac(6’)/aph(2’’)*, *aac(6’)/aph(2’’)*, *tetM*, *ermB*, and *tetM*-positive isolates were sensitive to VA, CN, S, DO, E, and T, respectively. Also, 77.8% of lincomycin-resistant isolates harbored the *ermB* gene. Statistical analysis revealed that there were significant differences in phenotypic-genotypic AMR for DO, E, T, and L, as illustrated in Supplementary Table 4.

### Distribution of class 1 and 2 integrons among enterococci isolates

48.1% and 3.7% of enterococci isolates harbored class 1 and 2 integrons, respectively, as illustrated in Table 6 and Supplementary Figs. 7 and 8. On comparison of the occurrence of class 1 integrons with the phenotypic and genotypic AMRPs of enterococci isolates, it was found that class 1 integrons-positive isolates showed more AMR and harbored more AMRGs, comparable with class 1 integrons-negative isolates, and statistical analysis revealed significant differences in the association between MDR and the *ermB* gene class 1 integrons, as illustrated in Table 7. Furthermore, 92.3% of class 1 integron-positive isolates were BFs. On the other hand, statistical analysis revealed that there were no significant differences in MDR- and AMRGs-class 2 integrons association, as illustrated in Supplementary Tables 5 and 6.

**Table 7:** Prevalence of multi-drug resistance and antimicrobial resistance genes among class 1 integrons-positive and negative enterococci isolates.

Presence of class 1 integrons	Multi-drug resistant enterococci (*n* = 17)		*aac(6’)/aph(2’’)* (*n* = 22)		ermB (*n* = 20)		*tetM* (*n* = 20)		vanA (*n* = 2)	
	No.	%	No.	%	No.	%	No.	%	No.	%
Class 1 integrons-positive isolates (n = 13)	12	70.6	12	54.5	13	65	12	60	2	100
Class 1 integrons- negative isolates (n = 14)	5	29.4	10	45.5	7	35	8	40	0	0
P value	0. 004^*^		0.326		0.006^*^		0.077		0.222	

## Discussion

The importance of enterococci in bird diseases, particularly poultry infection, has grown in recent years^40^. Contrary to Abed et al.^33^ and Hammam et al.^10^, who found that the prevalence of enterococci among the infected broiler chickens was 7.7% and 61.8%, respectively, enterococci were isolated from 22.5% of the examined broiler chickens in the present study. The infected birds displayed clinical signs and PM lesions similar to those found in enterococcal-infected broiler chickens by Stępień-Pyśniak et al.^41^ and Hammam et al.^10^.

*E*. *faecalis* and *E*. *faecium* are the most isolated *Enterococcus* spp. from poultry environment samples, but very few data are available about *Enterococcus* spp. isolated from the diseased poultry. Correct identification of enterococci is crucial for epidemiological and therapeutic purposes^41^. The VITEK 2 system is a reliable and fast technique for the identification of *Enterococcus* spp.^42^. Identification of enterococci isolates by the VITEK 2 system in this study revealed that *E*. *faecalis* is the most predominant species, followed by *E*. *faecium* and *E*. *gallinarum*, in agreement with the results of Stępień-Pyśniak et al.^41^, while it was in disagreement with those of Hammam et al.^10^, who found that *E*. *durans* was the most predominant species, followed by *E. faecalis*, without isolation of *E*. *faecium* or *E*. *gallinarum*. The variations in geographic location and breeding patterns may be the cause of the discrepancies in the prevalence of enterococci and their species^14,31^.

In vitro examinations for VFs and their associated genes, despite their limitations, provide useful insights into the actual virulence potential that the pathogens exhibit throughout the infection process^43^. In the present study, enterococci isolates were investigated for haemolytic activity, gelatinase activity, BF, and the presence of *cylA* and *gelE* genes by PCR, and most of the isolates had multiple of these VFs and genes (Table 3), which characterize the pathogenic isolates.

Enterococci secrete cytolysin and gelatinase, which are responsible for exacerbation of the infection. Enterococcal cytolysin is a hemolytic virulence factor linked to human diseases^44^. It was found that 28.6%, 11.1%, and 0% of *E. faecalis*,* E. faecium*, and *E. gallinarum* isolates were β-haemolytic, respectively. These results agreed with those of Diarra et al.^17^, who found that 28.6%, 10.4%, and 0% of *E. faecalis*,* E. faecium*, and *E. gallinarum* isolated from the commercial broiler farms were β-haemolytic, respectively, while these results were incompatible with those of Olsen et al.^36^ and Stępien-Pysniak et al.^37^, who found that all *E. faecalis* isolated from extra-intestinal lesions in poultry and yolk sac infections in broiler chicks were non-haemolytic, respectively. On the other hand, the *cylA* gene was detected in 42.9%, 33.3%, and 25% of *E. faecalis*,* E. faecium*, and *E. gallinarum* isolates, respectively, in disagreement with the results of Song et al.^45^, who didn’t detect the *cylA* gene in all *E. faecium* and *E. gallinarum* isolates and reported a lower prevalence of this gene (16%) among *E. faecalis* isolates. As previously reported by Olsen et al.^36^ and Stępien-Pysniak et al.^37^, haemolytic activity wasn’t detected among several *cylA*-positive enterococci isolates in this study, and this can be attributed to gene downregulation, presence of silent genes^37^, or to the fact that haemolytic activity requires the presence of the complete *cyl* operon (*cyl* L_L_L_S_ABM), which wasn’t investigated in this study^46^. Therefore, the haemolytic activity of enterococci should be determined by a combination of phenotypic and genotypic assays.

Gelatinase, encoded by the *gelE* gene, cleaves a broad range of host substrates and appears to contribute to BF^47,48^. All enterococci isolates in this study exhibited gelatinase activity and harbored the *gelE* gene. Our results nearly agreed with those of Olsen et al.^36^ and Stępien-Pysniak et al.^37^, who found that 95% and 98.7% of *E. faecalis* isolates exhibited gelatinase activity and harbored the *gelE* gene, respectively, while coming in contrast to those of Diarra et al.^17^, who found that all *E. faecium* and *E. gallinarum* isolates didn’t exhibit gelatinase activity or harbor the *gelE* gene. In the current study, gelatinase activity and *gelE* genes were reported in both biofilm and non-biofilm-forming *Enterococcus* isolates, confirming that gelatinase activity and *gelE* genes are important for the BF process, but many genetic factors and environmental conditions may be related to pathomechanism and BF by *Enterococcus* spp.^21^.

According to Wozniak-Biel et al.^49^, Enterococcal biofilm is of particular concern because it protects bacteria from host immunity, antimicrobials, and severe environmental conditions, which can make treatment and eradication of enterococci difficult. In this study, it was found that 85.7%, 88.9%, and 50% of *E. faecalis*,* E. faecium*, and *E. gallinarum* were BFs, respectively. These results agreed with the results of Ali et al.^43^, Stępien-Pysniak et al.^21^, and Alzahrani et al.^44^, who found that 50%, 83.3%, and 86.7% of *E. gallinarum*, *E. faecium*, and *E. faecalis* were BFs, respectively, while came disagreed with those of Diarra et al.^17^ and Wozniak-Biel et al.^49^, who found that all *Enterococcus* spp. were non-BFs and BFs, respectively.

MDR-enterococci attract attention^50^, and studies on antimicrobial susceptibility of enterococci have affirmed their worldwide emergence^51^. In this study, susceptibility of enterococci isolates was tested for 16 different antimicrobials, and they were entirely sensitive to VA and mostly sensitive to AMC, ENR, AX, CIP, AM, FF, CXM, SXT, and C, while they were mostly resistant to L, T, E, DO, S, and CN in agreement with findings of Obeng et al.^12^ while in contrast to the findings of Song et al.^45^ except in susceptibility of the isolates for VA, AM, and C. Furthermore, it was found that 63% of the isolates were MDR-enterococci in agreement with the findings of Bertelloni et al.^9^ and Maasjost et al.^52^ who reported that 61% and 61.4% of enterococci isolated from poultry flocks in Italy and Germany were MDR, respectively, while Kim et al.^53^ and Mwikuma et al.^14^ found that 39.4% and 90.5% of enterococci isolated from retail chicken meat in South Korea and from poultry in Zambia were MDR, respectively, and this could be attributed to numerous differences as geographical location, biosecurity and hygiene practices, as well as uses of antimicrobials^26,54^.

Unfortunately, these results may indicate the presence of high AMR among bacteria in broiler chickens, where enterococci are indicator bacteria widely used for monitoring of AMR^55^. The high prevalence of MDR-enterococci found in this study poses a hazard on poultry health and a potential public health concern as it limits the therapeutic options for enterococcal infections in poultry and humans. It may be related to the misuse of antimicrobials in poultry farms in the study area, where no laws or rules control the sale of antimicrobials^1^. As a result, antimicrobials must be prudently used and with the application of strict biosecurity measures.

VA resistance is considered the most serious enterococcal resistance^50^, where VA is the last resort for treatment of MDR-Enterococcal infections^56^. Furthermore, vancomycin-resistant *Enterococcus*, which has been reported worldwide, is an emerging main nosocomial pathogen in humans^57^, and its transmission from animals to humans has been reported^58^. Fortunately, in this study, all enterococci isolates were sensitive to VA in accordance with the results of Obeng et al.^12^, Maasjost et al.^52^, and Kwit et al.^59^, while Ahmed et al.^31^ found that 53.1% of *E. faecalis*, *E. faecium*, and *E. gallinarum* isolated from poultry in Egypt were vancomycin-resistant.

Regarding the molecular mechanism of *Enterococcus* spp. resistance to the different classes of antimicrobials, several genes were identified^49^. According to Chow^60^, Ayeni et al.^61^, Hajiahmadi et al.^62^, and Woz´niak-Biel et al.^49^, *aac*(*6’*)*/aph*(2′′), *tetM*, *vanA*, and *ermB* are the most common genes encoding high-level CN, T, VA, and macrolide resistance in enterococci, respectively. *Aac(6’*)*/aph*(2′′) also confer high-level resistance to amikacin, dibekacin, kanamycin, netilmicin, and tobramycin, but not to S^60^. In this study, 81.5%, 74.1%, 74.1%, and 7.4% of enterococci isolates harbored *aac*(*6’*)*/aph*(2′′), *ermB*, *tetM*, and *vanA* genes, respectively, in agreement with Woz´niak-Biel et al.^49^, who reported that 68.6%, 78.4%, and 11.8% of enterococci isolated from turkeys in Poland harbored *ermB*, *tetM*, and *vanA* genes, respectively, while Ayeni et al.^61^ reported that enterococci isolated from poultry in Nigeria didn’t harbor the *ermB* nor *vanA* gene, and only 23.3% of them harbored the *tetM* gene. *ermB* and *tetM* genes were found together in 66.7% of the isolates, and statistical analysis revealed the presence of a significant difference in their association (*P* = 0.001). Our findings agreed with the findings of Cauwerts et al.^19^, while Obeng et al.^12^ reported that only 20.6% of enterococci harbored the *ermB* and *tetM* genes together. AMRGs’ prevalence differences could be attributed to the different antimicrobials usage policy and strain geographical dispersion.

In this study, it was observed that the presence of AMRGs wasn’t necessarily related to their phenotypic expression in some enterococci isolates, where the phenotypic resistance wasn’t detected in all the isolates carrying the AMR gene, as illustrated in Table 6, and this could be attributed to the fact that these AMRGs were inactive^49^ or weren’t expressed in these isolates^19,49,63^ and also to resistance of enterococci to VA is conferred by different gene cluster aren’t investigated in this study^14^. This finding points to the importance of PCR as a gold standard test for the detection of AMR and the prevention of AMRGs spread. The *VanA* gene was reported in some enterococci isolates without displaying phenotypic VA resistance earlier by Getachew et al.^57^, Ribeiro et al.^64^ (2007) and Youssef et al.^30^. On the other hand, Ahmed et al.^32^, Cauwerts et al.^19^, and Woźniak-Biel, et al.^49^ detected the presence of *ermB*, *tetM*, and *aac*(*6’*)*/aph*(2′′) genes in some enterococci isolates susceptible to E, T, and CN, respectively. Also, 77.8% of lincomycin-resistant isolates harbored the *ermB* gene, which confers cross-resistance to macrolides, lincosamides, and streptogramin B group^37,41^.

Class 1 and 2 integrons have recently been detected in the clinical isolates of *E. faecalis* and *E*. *faecium*^62^, and to our knowledge, this is the first report about these integrons among the clinical enterococci isolates in Egypt. In this study, it was found that 48.1% and 3.7% of enterococci isolates harbored class 1 and 2 integrons, respectively. Our results agreed with the findings of Yayin et al.^65^ and Hajiahmadi et al.^62^, who found that 52.1% and 6.2% of *E. faecalis* and *E*. *faecium* harbored class 1 and 2 integrons, respectively, while Xu et al.^66^ and Noenchat et al.^55^ reported that 20% and 14.3% of *Enterococcus* spp. harbored class 2 and 1 integrons, respectively, and this could be attributed to the different sources of the isolates.

BF promotes the transfer of AMRGs carried on the integrons via the stress responses^67^. Results of this study showed a significant correlation between BF and the presence of the *int1* gene in enterococci isolates, where the *int1* gene was found with a much higher rate among BFs (92.3%) compared to non-BFs (7.7%), and this could be explained by the enhanced horizontal genetic exchange, including MGEs among the different species of bacteria in biofilm communities due to their closely packed structure^68^. In agreement with our findings, Alabdali^67^ found a similar association in enterococci isolates. Also, the significant correlation between BF and the presence of the *int1* gene was previously reported in other species of bacteria^69–72^.

Acquired AMR is the most important resistance of enterococci^73^, and enterococci can acquire high-resistance to an assortment of antimicrobials through horizontal transfer of MGEs^74^. AMRGs for all β-lactams and aminoglycosides, C, E, FF, L, rifampin, streptothricin, and trimethoprim were found in class 1 integrons^75^. The high prevalence of class 1 integrons found among enterococci isolates in this study, as well as the higher prevalence of the investigated AMRGs and MDR-enterococci reported among class 1 integrons-positive isolates, comparable with class 1 integrons-negative isolates (Table 7), may indicate the potential role of class 1 integrons in AMR of enterococci. However, we emphasize that to justify this preliminary inference and clarify the role of class 1 integrons in AMR of enterococci, additional studies with more detailed molecular analysis are required.

*Enterococcus* spp. are regarded as a potential source for the dissemination of AMRGs among bacteria^49^, and they are characterized by their relatively rapid dissemination through horizontal transfer of MGEs^73^, which is facilitated by biofilm structures^76^. Class 1 integrons act as a common mobile genetic element for the dissemination of many AMRGs among pathogens and commensals^77^. The high prevalence of MDR, BFs enterococci, which harbor several AMRGs and class 1 integrons, reported in this study (Tables 6 and 7), is of concern, as it may result in the spreading of AMRGs and development of MDR among other species of bacteria in poultry, and may human, complicating the prevention and control of bacterial diseases.

Despite providing valuable data on the prevalence of pathogenic *Enterococcus* spp. among broiler chickens in the study area, their AMR profiles, and the occurrence of class 1 and 2 integrons, this study has several limitations, primarily due to financial constraints. First, the sample size was relatively small and limited to two Governorates, which may restrict the generalizability of the findings. Therefore, larger-scale and longitudinal studies covering broader geographic regions are recommended to provide a more comprehensive investigation at the national level. Second, species-level identification of Enterococcus isolates was not confirmed by PCR, and antimicrobial susceptibility was not validated using MIC testing, which could have provided more precise characterization of resistance profiles. Finally, integron sequencing was not performed. Future studies should include sequencing analyses to characterize cassette content and to establish clearer links between integrons and AMR across different antimicrobial classes. Addressing these limitations in future research will help to strengthen the understanding of the epidemiology and public health significance of AMR of pathogenic *Enterococcus* spp. in poultry.

## Conclusion

This study demonstrated a high prevalence of pathogenic MDR-enterococci among broiler chickens at Sohag and Qena Governorates, Egypt, posing a hazard for poultry health and a potential public health concern. In addition, it revealed, to the best of our knowledge, for the first time in Egypt, that enterococci isolated from broiler chickens had a high prevalence of class 1 integrons. Therefore, strict biosecurity protocols and prudent antimicrobial stewardship strategies should be implemented urgently.

## Supplementary Information

Below is the link to the electronic supplementary material.

Supplementary Material 1

## Data Availability

The authors confirm that the data supporting the findings of this study are available within the article and its supplementary materials.

## Abbreviations

AM: Ampicillin
AMC: Amoxicillin/clavulanic acid
AMR: Antimicrobial resistance
AMRGs: Antimicrobial resistance genes
AMRPs: Antimicrobial resistance profiles
AX: Amoxicillin
BF: Biofilm formation
BFs: Biofilm formers
BHI: Broth brain heart infusion broth
C: Chloramphenicol
CIP: Ciprofloxacin
CN: Gentamicin
CXM: Cefuroxime
DO: Doxycycline
E: Erythromycin
E. faecalis: Enterococcus faecalis
E. faecium: Enterococcus faecium
E. gallinarum: Enterococcus gallinarum
ENR: Enrofloxacin
FF: Fosfomycin
L: Lincomycin
MDR: Multidrug-resistant
MGEs: Mobile genetic elements
OD: Optical density
S: Streptomycin
SXT: Trimethoprim/Sulphamethoxazole
T: Tetracycline
VA: Vancomycin
VFs: Virulence factors
