# Supplementary material for: Prevalence, antimicrobial resistance, and virulence factors of Enterococcus spp. infecting broiler chickens in Egypt
Source: Sci Rep. 2026 Jul 23;16:23070. doi: 10.1038/s41598-026-62799-3 (PMC13392404; doi:10.1038/s41598-026-62799-3)
Supplement: Supplementary file 1 — Supplementary Material 1 [file 41598_2026_62799_MOESM1_ESM.docx]

**Supplementary Figures and Figures Legend**


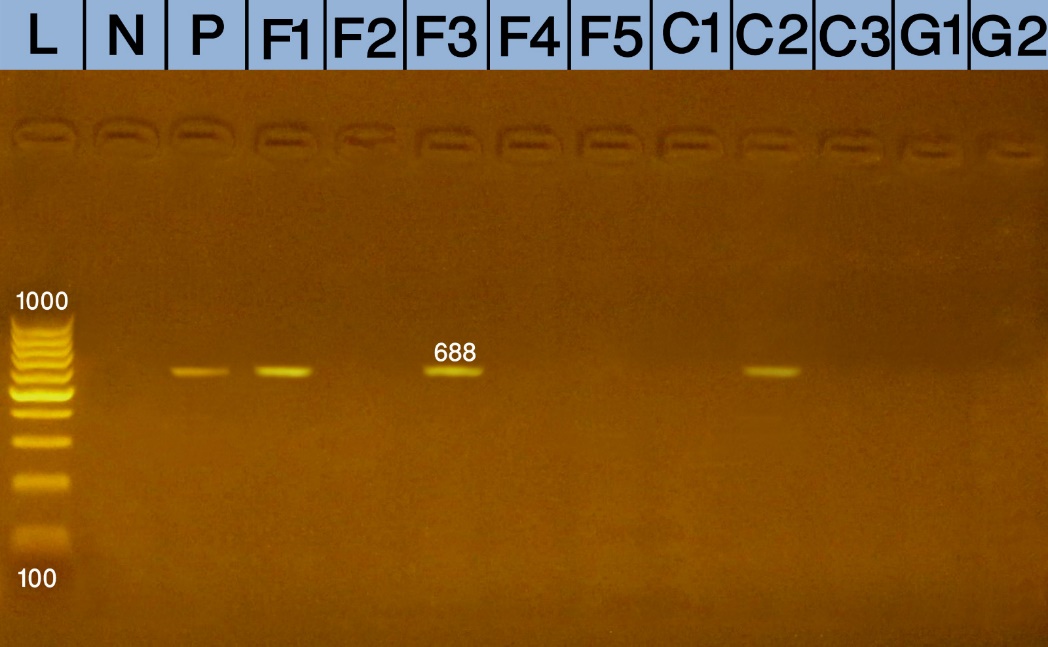


**Supplementary Fig. 1.** Agarose gel electrophoresis for PCR products that targeted *cylA* gene in enterococci isolates using specific primers. Lane L: DNA ladder (100 bp), lane P: Positive control, lane N: Negative control, and lanes F1-F5, C1-C3, and G1-G2: DNA extracted from *E. faecalis* isolates numbers 1-5, *E. faecium* isolates numbers 1-3, and *E. gallinarum* isolates numbers 1- 2, respectively, showing positive bands at 688 bp in isolates F1, F3, and C2 only.


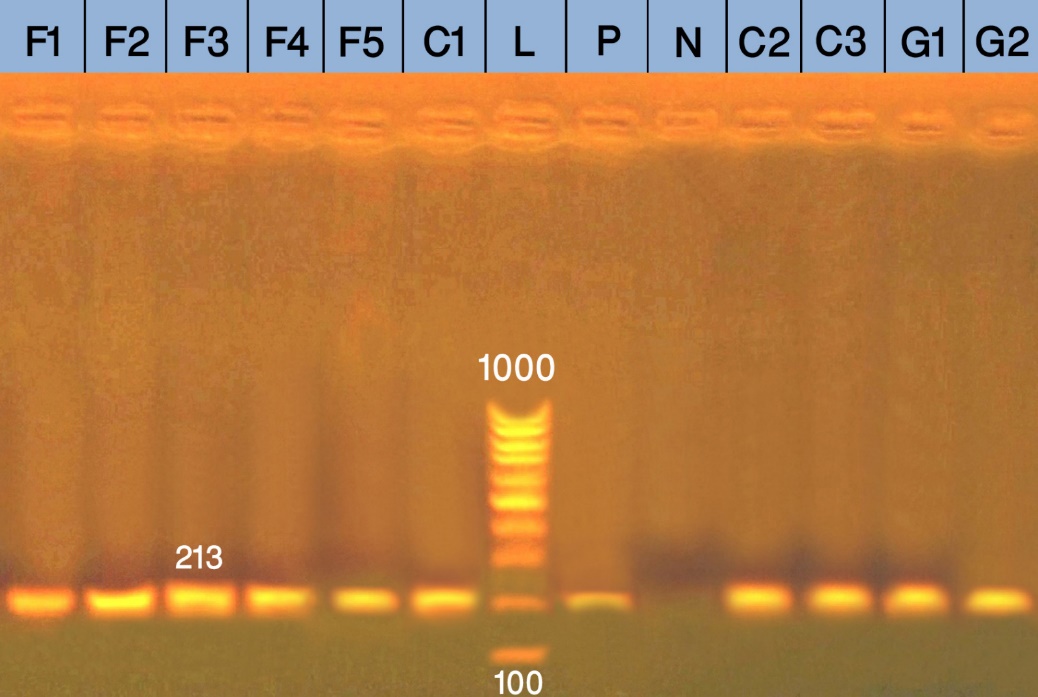


**Supplementary Fig. 2.** Agarose gel electrophoresis for PCR products that targeted *gelE* gene in enterococci isolates using specific primers. Lane L: DNA ladder (100 bp), lane P: Positive control, lane N: Negative control, and lanes F1-F5, C1-C3, and G1-G2: DNA extracted from *E. faecalis* isolates numbers 1-5, *E. faecium* isolates numbers 1-3, and *E. gallinarum* isolates numbers 1- 2, respectively, showing positive bands at 213 bp in all the isolates.


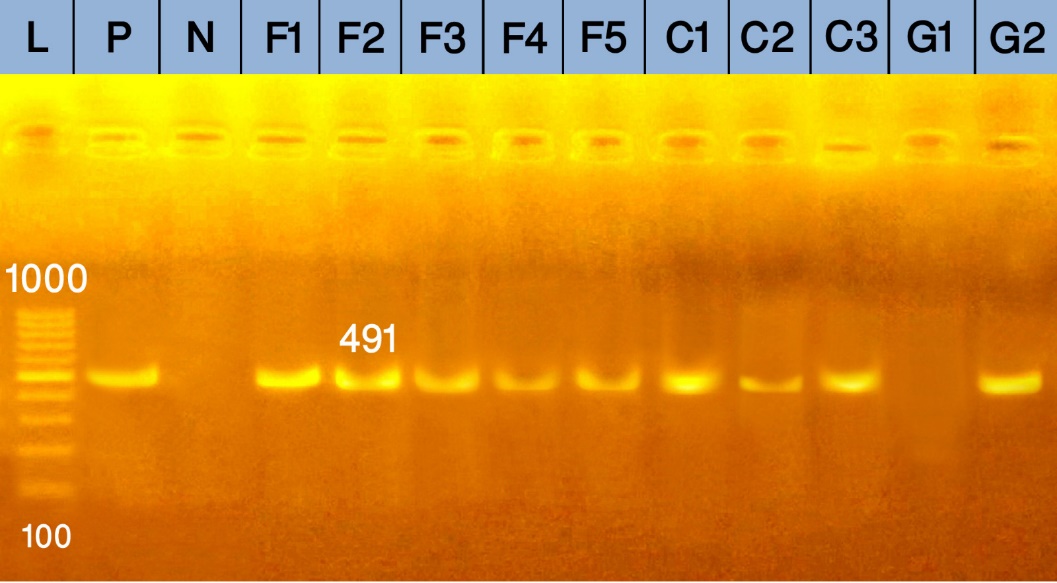


**Supplementary Fig. 3.** Agarose gel electrophoresis for PCR products that targeted *aac(6')/aph(2'')* gene in enterococci isolates using specific primers. Lane L: DNA ladder (100 bp), lane P: Positive control, lane N: Negative control, and lanes F1-F5, C1-C3, and G1-G2: DNA extracted from *E. faecalis* isolates numbers 1-5, *E. faecium* isolates numbers 1-3, and *E. gallinarum* isolates numbers 1- 2, respectively, showing positive bands at 491 bp in all the isolates except G1.


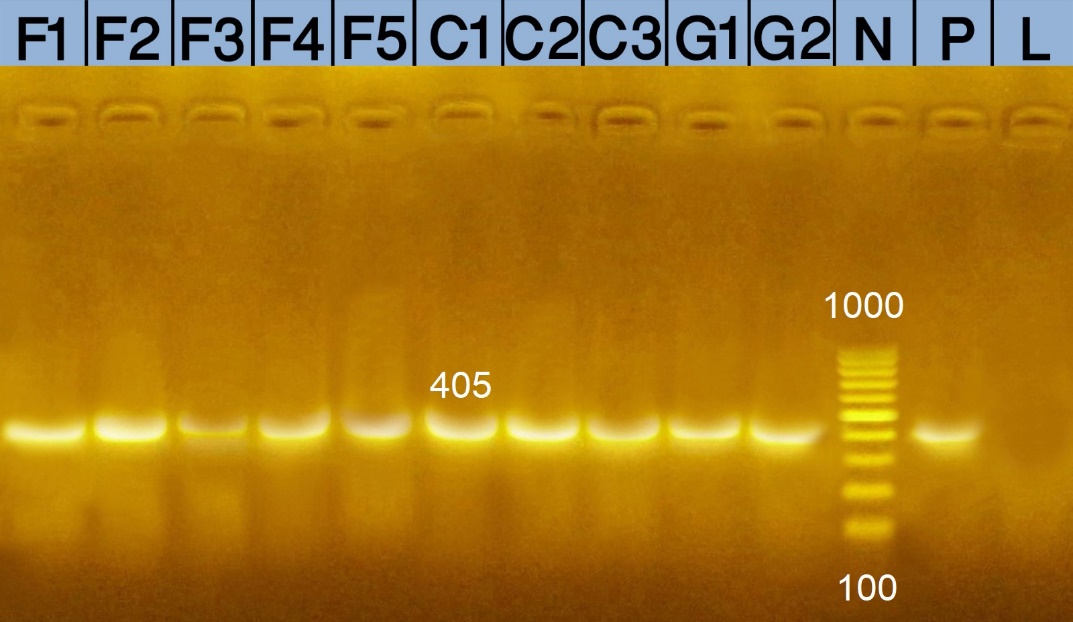


**Supplementary Fig. 4.** Agarose gel electrophoresis for PCR products that targeted *ermB* gene in enterococci isolates using specific primers. Lane L: DNA ladder (100 bp), lane P: Positive control, lane N: Negative control, and lanes F1-F5, C1-C3, and G1-G2: DNA extracted from *E. faecalis* isolates numbers 1-5, *E. faecium* isolates numbers 1-3, and *E. gallinarum* isolates numbers 1- 2, respectively, showing positive bands at 405 bp in all the isolates.


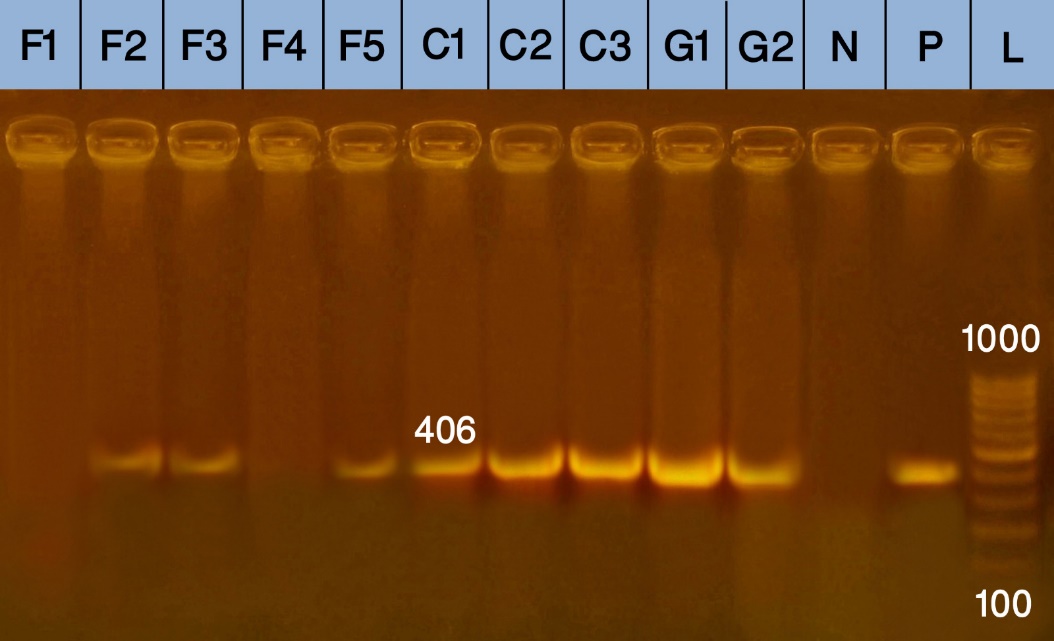


**Supplementary Fig. 5.** Agarose gel electrophoresis for PCR products that targeted *tetM* gene in enterococci isolates using specific primers. Lane L: DNA ladder (100 bp), lane P: Positive control, lane N: Negative control, and lanes F1-F5, C1-C3, and G1-G2: DNA extracted from *E. faecalis* isolates numbers 1-5, *E. faecium* isolates numbers 1-3, and *E. gallinarum* isolates numbers 1- 2, respectively, showing positive bands at 406 bp in all the isolates except F1 and F4.


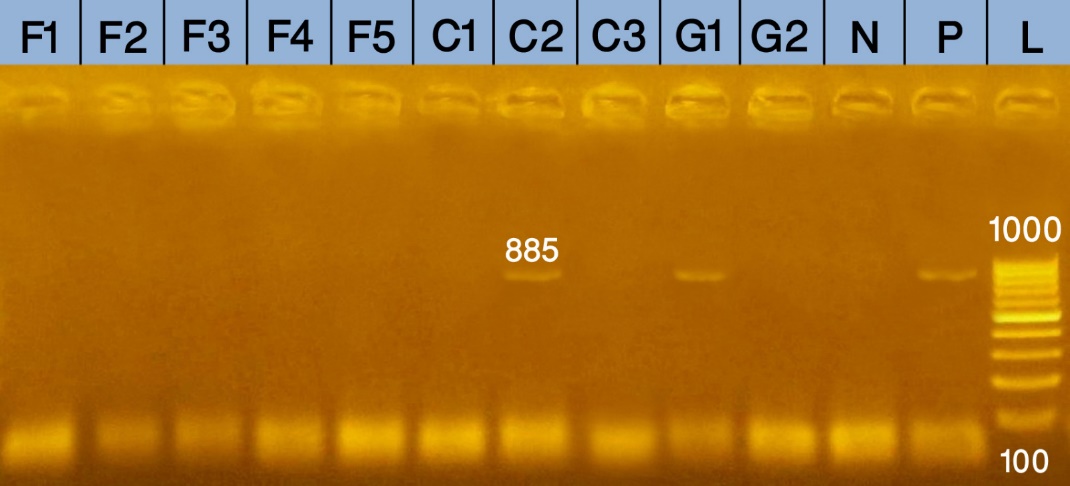


**Supplementary Fig. 6.** Agarose gel electrophoresis for PCR products that targeted *vanA* gene in enterococci isolates using specific primers. Lane L: DNA ladder (100 bp), lane P: Positive control, lane N: Negative control, and lanes F1-F5, C1-C3, and G1-G2: DNA extracted from *E. faecalis* isolates numbers 1-5, *E. faecium* isolates numbers 1-3, and *E. gallinarum* isolates numbers 1- 2, respectively, showing positive bands at 885 bp in isolates C2 and G1 only.


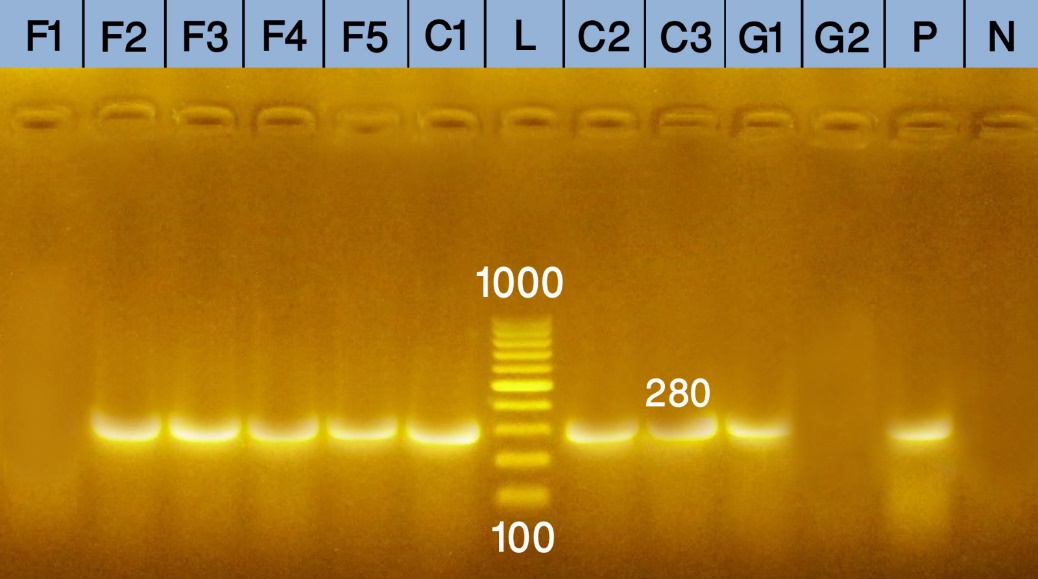


**Supplementary Fig. 7.** Agarose gel electrophoresis for PCR products that targeted *int1* gene in enterococci isolates using specific primers. Lane L: DNA ladder (100 bp), lane P: Positive control, lane N: Negative control, and lanes F1-F5, C1-C3, and G1-G2: DNA extracted from *E. faecalis* isolates numbers 1-5, *E. faecium* isolates numbers 1-3, and *E. gallinarum* isolates numbers 1- 2, respectively, showing positive bands at 280 bp in all the isolates except F1 and G2.


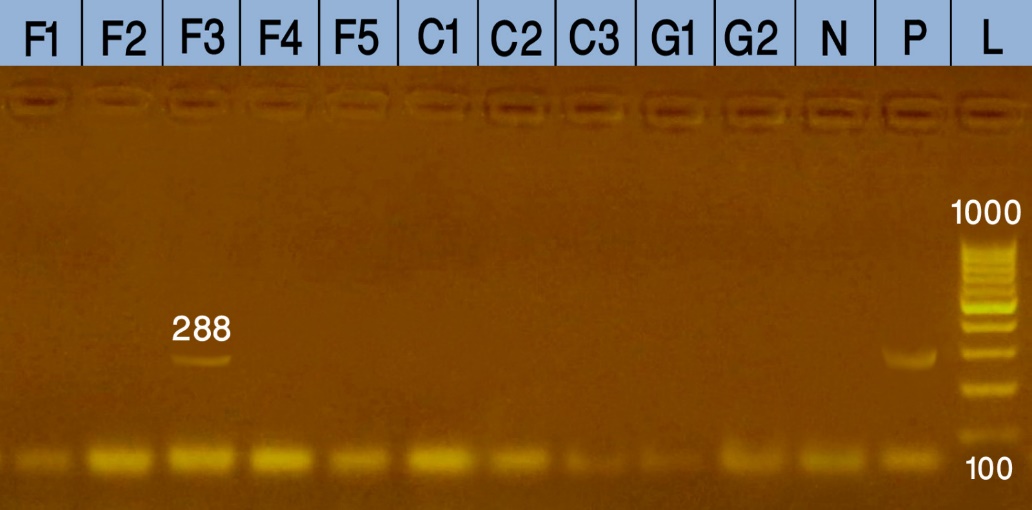


**Supplementary Fig. 8.** Agarose gel electrophoresis for PCR products that targeted *int2* gene in enterococci isolates using specific primers. Lane L: DNA ladder (100 bp), lane P: Positive control, lane N: Negative control, and lanes F1-F5, C1-C3, and G1-G2: DNA extracted from *E. faecalis* isolates numbers 1-5, *E. faecium* isolates numbers 1-3, and *E. gallinarum* isolates numbers 1- 2, respectively, showing positive bands at 288 bp in isolate F3 only.

**Supplementary Tables**

**Supplementary Table 1** Phenotypic antimicrobial resistance and biofilm formation association (*n*=27).

| **Antimicrobial Agent** | **Phenotypic resistance** | **Biofilm formation** | | | | **P value** |
| --- | --- | --- | --- | --- | --- | --- |
|  |  | **Non-biofilm formers (n)** | **Weak biofilm formers (n)** | **Moderate biofilm formers (n)** | **Strong biofilm formers (n)** |  |
| AM | Sensitive  Intermediate  Resistant | 5  0  0 | 2  0  0 | 11  1  1 | 2  2  3 | 0.101 |
| AX | Sensitive  Intermediate  Resistant | 5  0  0 | 2  0  0 | 12  1  0 | 4  0  3 | 0.084 |
| AMC | Sensitive  Intermediate  Resistant | 5  0  0 | 2  0  0 | 13  0  0 | 4  1  2 | 0.089 |
| CXM | Sensitive  Intermediate  Resistant | 5  0  0 | 1  1  0 | 8  2  3 | 2  2  3 | 0.254 |
| VA | Sensitive  Intermediate  Resistant | 5  0  0 | 2  0  0 | 13  0  0 | 7  0  0 | **-** |
| E | Sensitive  Intermediate  Resistant | 1  1  3 | 0  0  2 | 2  3  8 | 1  2  4 | > 0.999 |
| CN | Sensitive  Intermediate  Resistant | 1  1  3 | 1  0  1 | 4  3  6 | 2  1  4 | > 0.999 |
| S | Sensitive  Intermediate  Resistant | 0  1  4 | 1  0  1 | 4  3  6 | 1  2  4 | 0.785 |
| CIP | Sensitive  Intermediate  Resistant | 5  0  0 | 2  0  0 | 10  2  1 | 2  3  2 | 0.175 |
| ENR | Sensitive  Intermediate  Resistant | 5  0  0 | 2  0  0 | 10  2  1 | 2  4  1 | 0.123 |
| C | Sensitive  Intermediate  Resistant | 3  2  0 | 0  1  1 | 7  3  3 | 1  1  5 | 0.109 |
| T | Sensitive  Intermediate  Resistant | 0  3  2 | 0  1  1 | 4  1  8 | 0  0  7 | **0.034^*^** |
| DO | Sensitive  Intermediate  Resistant | 1  2  2 | 1  0  1 | 5  1  7 | 0  1  6 | 0.242 |
| L | Sensitive  Intermediate  Resistant | 1  2  2 | 0  0  2 | 4  1  8 | 0  1  6 | 0.362 |
| FF | Sensitive  Intermediate  Resistant | 4  1  0 | 2  0  0 | 7  4  2 | 1  3  3 | 0.271 |
| SXT | Sensitive  Intermediate  Resistant | 5  0  0 | 1  1  0 | 8  2  3 | 1  1  5 | **0.031^*^** |

**Supplementary Table 2** Multi-drug resistance and biofilm formation association (*n*=27).

| **Multi-drug resistance** | **Biofilm formation** | | | | **P value** |
| --- | --- | --- | --- | --- | --- |
|  | **Non-biofilm formers (n)** | **Weak biofilm formers (n)** | **Moderate biofilm formers (n)** | **Strong biofilm formers**  **(n)** |  |
| Multi-drug resistant isolate  Non Multi-drug resistant isolate | 2  3 | 2  0 | 7  6 | 6  1 | 0.298 |

**Supplementary Table 3** *ermB* and *tet* (M) genes association (*n*=27).

| ***ermB* gene** | ***tetM* gene** | | **P value** |
| --- | --- | --- | --- |
|  | **(-) (n)** | **(+) (n)** |  |
| *ermB* (+)  *ermB* (-) | 2  5 | 18  2 | **0.005^*^** |

**Supplementary Table 4** Phenotypic-genotypic antimicrobial resistance association (*n*=27).

| **Antimicrobial agent** | **Genotypic resistance** | **Phenotypic resistance** | | | **P value** |
| --- | --- | --- | --- | --- | --- |
|  |  | **Sensitive (n)** | **Intermediate (n)** | **Resistant (n)** |  |
| CN | *aac(6')/aph(2'')* (+) | 5 | 4 | 13 | 0.221 |
|  | *aac(6')/aph(2'')* (-) | 3 | 1 | 1 |  |
| S | *aac(6')/aph(2'')* (+) | 3 | 5 | 14 | 0.060 |
|  | *aac(6')/aph(2'')* (-) | 3 | 1 | 1 |  |
| E | *ermB* (+) | 0 | 3 | 17 | **< 0.001^*^** |
|  | *ermB* (-) | 4 | 3 | 0 |  |
| L | *ermB* (+) | 5 | 1 | 14 | **0.043^*^** |
|  | *ermB* (-) | 0 | 3 | 4 |  |
| DO | *tetM* (+) | 1 | 3 | 16 | **< 0.001^*^** |
|  | *tetM* (-) | 6 | 1 | 0 |  |
| T | *tetM* (+) | 1 | 2 | 17 | **0.002^*^** |
|  | *tetM* (-) | 3 | 3 | 1 |  |
| VA | *vanA* (+) | 2 | 0 | 0 | - |

**Supplementary Table 5** Multi-drug resistance and class 2 integrons association (*n*=27).

| **Multi-drug resistant** | ***int2* gene** | | **P value** |
| --- | --- | --- | --- |
|  | **(-) (n)** | **(+) (n)** |  |
| Multi-drug resistant isolate  Non Multi-drug resistant isolate | 16  10 | 1  0 | > 0.999 |

**Supplementary Table 6** Genotypic antimicrobial resistance and class 2 integron association (*n*=27).

| **Antimicrobial resistance gene** | ***int2* gene** | | **P value** |
| --- | --- | --- | --- |
|  | **(-) (n)** | **(+) (n)** |  |
| *aac(6')/aph(2'')* (+)  *aac(6')/aph(2'')* (-) | 21  5 | 1  0 | > 0.999 |
| *ermB* (+)  *ermB* (-) | 19  7 | 1  0 | > 0.999 |
| *tetM* (+)  *tetM* (-) | 19  7 | 1  0 | > 0.999 |
| *vanA* (+)  *vanA* (-) | 2  24 | 0  1 | > 0.999 |
